# Hydrological controls on base metal precipitation and zoning at the porphyry-epithermal transition constrained by numerical modeling

**DOI:** 10.1038/s41598-023-30572-5

**Published:** 2023-03-07

**Authors:** Malte Stoltnow, Philipp Weis, Maximilian Korges

**Affiliations:** 1 Institute of Earth and Environmental Science, University of Potsdam, Karl-Liebknecht-Straße 24/25, 14476 Potsdam, Germany; 2 GFZ German Research Centre for Geosciences, Telegrafenberg, 14473 Potsdam, Germany

**Keywords:** Geochemistry, Hydrogeology, Mineralogy, Solid Earth sciences

## Abstract

Ore precipitation in porphyry copper systems is generally characterized by metal zoning (Cu–Mo to Zn–Pb–Ag), which is suggested to be variably related to solubility decreases during fluid cooling, fluid-rock interactions, partitioning during fluid phase separation and mixing with external fluids. Here, we present new advances of a numerical process model by considering published constraints on the temperature- and salinity-dependent solubility of Cu, Pb and Zn in the ore fluid. We quantitatively investigate the roles of vapor-brine separation, halite saturation, initial metal contents, fluid mixing and remobilization as first-order controls of the physical hydrology on ore formation. The results show that the magmatic vapor and brine phases ascend with different residence times but as miscible fluid mixtures, with salinity increases generating metal-undersaturated bulk fluids. The release rates of magmatic fluids affect the location of the thermohaline fronts, leading to contrasting mechanisms for ore precipitation: higher rates result in halite saturation without significant metal zoning, lower rates produce zoned ore shells due to mixing with meteoric water. Varying metal contents can affect the order of the final metal precipitation sequence. Redissolution of precipitated metals results in zoned ore shell patterns in more peripheral locations and also decouples halite saturation from ore precipitation.

## Introduction

Porphyry copper systems are our main resources for the global supply of Cu and in addition contain a large variety of other metals at economic or sub-economic grades^1^. Ore precipitation in porphyry-centered magmatic-hydrothermal systems generally produces base metal zoning patterns that transition upwards and outwards from proximal Cu–Mo to distal Zn–Pb–Ag, with variable lateral extents of up to 8 km^1,2^. Experimental studies and thermodynamic modeling indicate that cooling of magmatic fluids accompanied by fluid-rock interaction is a first-order control on metal precipitation and zonation patterns, resulting from different solubilities of the respective metals along fluid pathways^3,4^. The timing and role of incursion of external fluids for ore formation are debated, with some studies indicating that fluid mixing may already occur during primary mineralization and/or that later circulation can cause metal redistribution^5–8^.

Metals such as Cu, Pb and Zn in porphyry systems are predominantly transported by hydrothermal fluids as chloride complexes under elevated temperatures and rather acidic conditions^9,10^. Fluid inclusion data suggest that fluids exsolving from granitic to granodioritic intrusions in the upper crust yield bulk salinities of 5 to 15 wt% NaCl_equiv_^11^. Depending on the fluid salinity, temperature, pressure and the metal content of the parental magma, metal contents of the primary single-phase magmatic fluid vary between 20 and 20,000 ppm Cu (mean 2660 ppm), 10 and 4500 ppm Pb (mean 330 ppm), as well as 20 and 6500 ppm Zn (600 ppm)^12^.

This primary fluid exsolved from the magma phase-separates upon ascent due to decompression into a low-salinity vapor and a hypersaline liquid (brine) phase. Fluid inclusion analyses and experimental studies show that base metals like Cu, Pb and Zn preferentially partition into the brine phase^13^, with apparent Cu partitioning into the vapor phase now being explained as an artifact due to post-entrapment diffusion into vapor inclusions^14,15^. However, the respective roles of the vapor and brine phases for mineralization in porphyry Cu systems remain debated, because mass balance considerations suggest that phase separation at depth produces larger amounts of vapor than brine^16,17^ and some of the ascending vapor phase can condense into a liquid phase during ascent and cooling^18–20^. Geophysical evidence and numerical modeling further suggest that brine lenses form beneath active and dormant volcanoes^21^, which inspired propositions that these metal-rich hypersaline fluids with Cu contents of up to 7000 ppm stored at depth may have an economic potential^22^. However, it remains unknown whether such brine accumulations are long-lived or rather transient features, with both scenarios being permissive in different set-ups in numerical simulations^22,23^.

Furthermore, a large number of ore-stage brine inclusions homogenize by halite disappearance, which either reflects saturation in solid halite^24^ or post-entrapment modifications^25^. Halite saturation also occurs in numerical simulations of porphyry systems using different models and set-ups^26,27^. The transition from liquid–vapor stability toward vapor-halite stability of the ascending fluid would be accompanied by an abrupt decrease and eventually disappearance of the liquid mass fraction. This process would lead to an abrupt decrease in the capacity of the fluid to carry metals in solution, which could lead to base metal precipitation^24^, albeit further transportation as dispersed particles during potential eruptive processes at these conditions may also be possible.

Quantifying the dynamic behavior of mass and heat transfer in porphyry systems can be approached by numerical simulations using models that can handle high-temperature multi-phase flow of H_2_O–NaCl fluids in porous media^28^. These numerical models can simulate the evolution of the mineralization and have provided insightful results for specific ore-forming processes^26,29^, but still rely on some simplifications. In particular, capabilities for full reactive-transport modeling including chemical speciation, pH and redox conditions as well as the role of ligand complexing for metal transport in the formation of high-temperature ore deposits are still under development. Previous work using the software CSMP++ focused on the physical hydrology and implemented a simple proxy for temperature-dependent Cu enrichment^23,26,29^. These numerical simulations suggest that the dimensions of the resulting ore shell depend on the interplay between magmatic fluid release, meteoric water convection and dynamic permeability distributions, reproducing typical copper ore bodies with bell-like shapes and lateral extents of 1–3 km ^23^. Recent simulations of clastic-dominant (CD-type) Pb–Zn deposits using the same model further considered the remobilization of Pb and Zn by circulating heated fluids, but based their parameterizations only on the temperature dependence of metal solubility^30^. These models do not yet consider the effect of fluid salinity and metal partitioning between fluid phases.

For this study, we therefore developed a new parametrization, which considers individual temperature- and salinity-dependent solubilities for Cu, Pb and Zn. The used metal solubilities are based on calculations of Kouzmanov and Pokrovski^12^, similar but not identical to parameterizations in the simulations of Blundy, et al.^22^, which also introduced temperature- and salinity-dependent Cu solubilities and partitioning during phase separation to their model. The addition of Pb and Zn provides a further constraint for the hydrology at the porphyry-epithermal transition and an opportunity to test whether metal zoning with variable lateral extents can be explained by simple cooling along characteristic fluid pathways. Furthermore, we use the remobilization functionality introduced by Rodríguez, et al.^30^ to constrain its impact on base metal ore shells related to porphyry systems. To date, a full representation of fluid-rock interactions at these conditions is not yet possible, because it would require an internally consistent thermodynamic model that can reliably cover high-temperature, low-density fluid phases. However, the new developments mark a step forward towards more geological realism by including additional geochemical constraints. We use this augmented numerical model to investigate the role of phase separation, brine formation, halite saturation, fluid mixing and remobilization as first-order controls of the physical hydrology on precipitation mechanisms of base metals in porphyry systems.

## Methods

### Governing equations

We calculate fluid flow with a continuum approach for porous media using the Control Volume Finite Element Method (CVFEM) numerical scheme which is implemented in the Complex Systems Modeling Platform (CSMP + +)^31^. Using a realistic expression of the nonlinear fluid properties (e.g., density, viscosity and enthalpy) and the physical separation of fully miscible multi-phase fluids (liquid, vapor and halite in the H_2_O–NaCl system), the model can perform compressible fluid flow at temperatures up to 1000 °C and pressures up to 500 MPa^32,33^.

Given the Darcy velocity $v$ of a fluid phase i (liquid (l) or vapor (v)), using the extended form of Darcy's law, the circulation of these fluids is calculated as

1 $$ v_{i} = - k\frac{{k_{r,i} }}{{\mu_{i} }}\left( {\nabla p - \rho_{i} g} \right), i = l,v $$

where $k$ is the bulk rock permeability and ${k}_{r,i}$ the relative permeability, ${\mu }_{i}$ the dynamic viscosity and ${\rho }_{i}$ the density of the indicated fluid phase $i$. The gravitational acceleration is given by $g$ and the total fluid pressure by $p$^34^. Saline fluids may further precipitate a solid halite phase $h$. We use a linear relative permeability model with

2 $$ k_{rv} + k_{rl} = 1 - S_{h} $$

where ${S}_{h}$ is the volumetric saturation of the immobile halite phase. The residual saturation of the liquid is given as ${R}_{l}=0.3(1-{S}_{h})$ and the residual saturation of the vapor as ${R}_{l}=0.0$.

The conservation of fluid mass is calculated as

3 $$ \frac{{\partial \left( {\phi \left( {S_{l} \rho_{l} + S_{v} \rho_{v} + S_{h} \rho_{h} } \right)} \right)}}{\partial t} = - \nabla \left( {v_{l} \rho_{l} } \right) - \nabla \left( {v_{v} \rho_{v} } \right) + Q_{{H_{2} O + NaCl}} $$

where $\phi$ is the porosity, $Q_{{H_{2} O + NaCl}}$ refers to the source term of fluid mass and $t$ is the time. The conservation of salt mass is given by

4 $$ \frac{{\partial \left( {\phi \left( {S_{l} \rho_{l} X_{l} + S_{v} \rho_{v} X_{v} + S_{h} \rho_{h} } \right)} \right)}}{\partial t} = - \nabla \left( {v_{l} \rho_{l} X_{l} } \right) - \nabla \left( {v_{v} \rho_{v} X_{v} } \right) + Q_{NaCl} $$

with ${Q}_{NaCl}$ as the source term and ${X}_{i}$ as the mass fraction of NaCl in the indicated fluid phase. The conservation of energy is described by heat advection through the fluid and heat transfer through the rock with the thermal conductivity $K$ and is given by

5 $$ \frac{{\partial (\left( {1 - \phi } \right) {\rho_{r} h_{r} + \phi \left( {S_{l} \rho_{l} h_{l} + S_{v} \rho_{v} h_{v} + S_{h} \rho_{h} h_{h} } \right))}}} {\partial t} = \nabla \left( {K\nabla T} \right) - \nabla \left( {v_{l} \rho_{l} h_{l} } \right) - \nabla \left( {v_{v} \rho_{v} h_{v} } \right) + Q_{e} $$

where the rock is denoted by the subscript $r$ and ${h}_{i}$ is the specific enthalpy of the phase $i$. The heat source term is given as ${Q}_{e}$ and the temperature as $T$. Assuming local thermal equilibrium between fluid and rock, the total enthalpy within a control volume of the mesh is distributed over the rock and fluid based on their thermodynamic properties.

The permeability $k$ is modeled as a dynamic element property^23,26^ and is based on the following assumptions: (1) a depth-dependent background permeability profile, (2) a near-critically stressed brittle crust and a consequential failure criterion for fractures at near-hydrostatic fluid pressure conditions, (3) an increasingly ductile behavior through heating, leading to a reduction in permeability and differential stress, causing failure criteria at near-lithostatic fluid pressures (with the transition from brittle to ductile starting at 360 °C), (4) a pressure-dependent permeability increase, which counteracts the temperature-dependent permeability decrease and (5) an incremental increase in permeability in response to hydraulic fracturing by as much as two orders of magnitude if fluid pressures exceed the local stress-state-dependent failure criterion. Further details on the numerical approach and benchmark solutions are given in Weis^26^ and Weis, et al.^31^.

### Proxies for metal transport, partitioning, Tx-dependent solubilities and precipitation

The main focus of this study is to advance the numerical model for porphyry Cu systems with respect to (i) the solubility of Cu, Pb and Zn in the ore fluid in dependence on temperature and salinity, (ii) the partitioning of these metals between vapor and liquid, (iii) the metal transport by ore fluids, (iv) varying metal concentrations of the primary magmatic fluid and resulting metal enrichment potential and (v) remobilization of previously precipitated metals.

**Figure 1:**
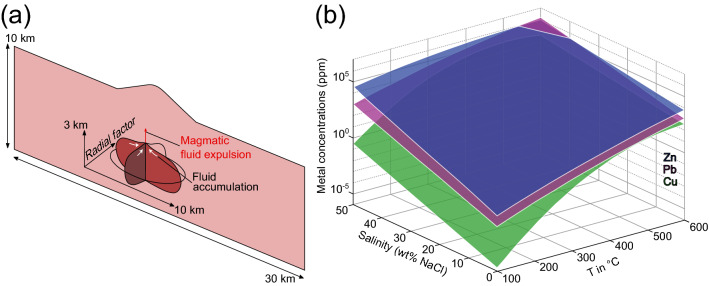
Model configuration and proxies for metal solubilities (**a**) The modeling domain represents a two-dimensional cross-section of the upper crust with dimensions of 10 × 30 km in height and width, including a magma reservoir with an extension of 3 × 10 km. The radial factor describes the extent of the magma chamber in the (unresolved) third dimension relative to the extent in the horizontal direction and thereby controls the total volume of the magma reservoir in three dimensions. Magmatic fluids are assumed to accumulate beneath a cupola region at about 5 km depth before release to the host rock (modified from Weis^26^). (**b**) Modeled proxies for temperature- and salinity-dependent metal solubilities based on thermodynamic calculations by Kouzmanov and Pokrovski ^12^ for zinc, lead and copper.

i) Metal solubilities are calculated using temperature- and salinity-dependent functions for the individual metals ${C}_{i}(T,x)$ applying a simple interpolation of the pyrite–magnetite–haematite-saturated solubility data at pH = 5 of Kouzmanov and Pokrovski^12^ for elevated temperatures and salinities with

6 $$ \log \left( {C_{i} } \right) = a_{00,i} + a_{10,i} T + a_{01,i}  x + a_{20,i} T^{2} + a_{11,i}  x T $$

where T is the temperature in °C, x is the weight fraction NaCl_eq_ in the fluid, a_00,Cu_ = −9.215, a_01,Cu_ = 0.1126, a_10,Cu_ = 0.03875, a_20,Cu_ = −3.209 ∙ 10^–5^, a_11,Cu_ = −1.213 ∙ 10^–4^, a_00,Pb_ = −3.269, a_01,Pb_ = 0.1068. a_10,Pb_ = 0.01408, a_20,Pb_ = −5.685 ∙ 10^–6^, a_11,Pb_ = −7.534 ∙ 10^–5^, a_00,Zn_ =  − 2.234, a_01,Zn_ = 0.1245, a_10,Zn_ = 0.01313, a_20,Zn_ = −4.914 ∙ 10^–6^, a_11,Zn_ = −1.367 ∙ 10^–4^. The resulting expressions are shown in Fig. 1b. These solubility relationships must be refined with further experimental data for T > 400 °C and salinities > 40 wt% NaCl_equiv_ as they are not yet constrained by the data from Kouzmanov and Pokrovski^12^. For the time being, we use extrapolations to higher temperatures and salinities with this parameterization, which leads to higher solubilities in these ranges and is generally in line with other studies on Cu contents in hydrothermal fluids^14^. However, metal precipitation is expected to rather occur within the data range covered in Kouzmanov and Pokrovski^12^. Metals are completely dissolved in the fluid phases if ${c}_{t}\le {c}_{metal}(T,x)$ and metals are precipitated when ${c}_{t}>{c}_{eq}(T,x)$. The capacity to form sulfide minerals typical for porphyry systems (e.g., Cu: chalcopyrite, bornite, chalcocite; Pb: galena; Zn: sphalerite) would depend on sulfur availability and other chemical parameters such as pH and redox, which cannot be resolved here.

ii) The total metal concentrations, ${C}_{t}$, in vapor and liquid phases are expressed by

7 $$ C_{t} = \mathop \sum \limits_{i = g,l} \rho_{i} C_{i} S_{i} \left/\mathop \sum \limits_{i = g,l} \rho_{i} S_{i}\right. $$

Analytical and experimental data indicate that metal partitioning between liquid and vapor depends on the salinity of the respective phase. We approximate this observation by first calculating the solubility of the individual phases with their respective salinities and then calculate the solubility of the bulk fluid. If the fluid is saturated in a respective metal, we partition the concentration according to the respective phase solubilities, leading to a salinity- and mass-dependent partitioning. If the bulk fluid is undersaturated in a metal, we use the same degree of partitioning by assuming the same degree of undersaturation in both phases.

iii) We introduce Cu, Pb and Zn as tracers to the mass conservation as

8 $$ \frac{{\partial \left( {\phi \left( {S_{l} \rho_{l} C_{l} + S_{v} \rho_{v} C_{v} } \right)} \right)}}{\partial t} = - \nabla \cdot \left( {v_{l} \rho_{l} C_{l} } \right) - \nabla \cdot \left( {v_{v} \rho_{v} C_{v} } \right) + \nabla \cdot \left( {D_{v} \nabla \left( {\rho_{l} C_{l} } \right)} \right) + Q_{i} $$

with the individual metal concentration in the fluid ${C}_{i}$, the diffusion–dispersion coefficient *D*, and a source term ${Q}_{i}= {C}_{i}^{initial} \cdot {Q}_{{H}_{2}O+NaCl}$, reflecting varying Cu, Pb and Zn concentrations ${C}_{i}^{initial}$ in the primary magmatic ore fluid. For simplicity, this formulation assumes that no metals are stored in the solid halite phase. In our simulations the amount of fluid expelled from the magma chamber depends on the size of the magma chamber, which is controlled by the radial factor (Fig. 1a). The radial factor describes the extension of the magma chamber in the unresolved z direction of a two-dimensional modeling domain. Given an extension of 10 km in x direction, a radial factor of 0.5 would correspond to an extension of 5 km in z direction (Fig. 1a).

iv) To ensure the best possible comparability with other numerical modeling studies^23,26,29^, the primary magmatic fluid contains 500 ppm Cu, which is based on measurements of intermediate density (ID) fluid inclusions in porphyry Cu systems and experimental studies. The Pb concentrations of 330 ppm as well as Zn concentrations of 600 ppm refer to mean values of ID fluid inclusions originating from numerous porphyry Cu deposits^12^. To study the effect of the initial metal concentrations and metal zoning, we also performed simulations with lower initial Zn concentrations of 330 ppm (same as Pb) and Pb concentrations of 33 ppm. As a modeling proxy for ore grade distributions, we calculate metal enrichment potentials as

9 $$ \psi_{i} = \frac{{m_{i} }}{{C_{i}^{initial} C_{water} \rho_{rock} V}} $$

with the mass of the precipitated metals ${m}_{i}$ and the control volume $V.$

v) In a first suite of simulations, we consider that metals only accumulate over time due to metal precipitation from oversaturated fluids. As an additional functionality, we include remobilization of previously precipitated metals if undersaturated hydrothermal fluids flow through volumes containing metal precipitates. We assume that fluids can remobilize metals until either the fluids are saturated or the respective volume is fully depleted in the respective metal. This functionality for remobilization also covers the potential case that Cu, Pb and Zn are temporarily stored as Cl-complexes together with solid halite and also get subsequently redissolved by later ingression of less saline fluids. Further downstream, the metals can get reprecipitated as soon as the fluids become oversaturated again.

Although we account for temperature- and salinity-dependent metal solubilities, the current model cannot resolve the effect of chemical speciation (e.g. incl. pH, redox, chloride and bisulfide complexing) on metal transport, partitioning, precipitation and remobilization. Full reactive-transport has only been well established for less complex hydrothermal systems forming at lower temperatures^35–37^, but has the potential to be extended to higher temperatures once robust thermodynamic datasets are available. For the time being, we have to use the simplifying assumptions mentioned above and critically consider them when evaluating the modeling results.

### Model configuration

The modeling domain is a two-dimensional cross-section of 30 km width and 10 km height and an additional topography described by a central volcano of about 1.5 km height (Fig. 1a). An elliptical magma chamber (~ 3 km height, 10 km width) with an initial temperature of 900 °C is emplaced at 5 km depth (Fig. 1a). Previous modeling work resolving incremental magma growth shows that such magma reservoirs can be built up with magma fluxes in the order of about 10^−2^ km^3^/y^29^. Two radial factors (0.25 and 0.5) were applied resulting in a theoretical magma volume of ~ 47 km^3^ and ~ 95 km^3^, respectively. This is in line with a presumed minimal volume of 50 km^3^ proposed to form porphyry Cu deposits^1,38^. An approximately doubled magma volume is also accompanied by an approximately doubled fluid release rate, with primary magmatic fluids assumed to be expelled exclusively from the cupola region of the magma chamber (Fig. 1a). Previous modeling work resolving magma degassing mechanisms from similarly sized reservoirs describes how focused fluid release can self-organize during cooling and degassing^39^. With this configuration, fluid release is directly related to the cooling and crystallization of the magma reservoir due to heat conduction and convection of surrounding fluids^23^. This parameterization leads to fluid release once the outer rim has reached water saturation, with highest release rates in the earlier stages and a gradual decline as the reservoir undergoes radial cooling.

Previous studies using this model for porphyry copper systems have highlighted how the dynamic permeability evolution controls the thermal evolution^23^. Overall higher permeabilities lead to spatially more confined potential ore grades. Similarly, porphyry stocks that are also injected above the cupola region in natural systems but are neglected in our simplified geometry may also serve as higher-permeability pathways that could support fluid focusing. Further, the convection of surrounding meteoric fluids at depth generally flows along the margins of the magma reservoir towards the cupola region. However, the topography of a stratovolcano leads to lateral transport of mixed magmatic-meteoric fluids at shallower levels towards the base of the volcano due to the hydraulic head of the groundwater column^26^. Even though the simulations with moderate host rock permeability underneath a stratovolcano produce less confined porphyry copper ore shells, we use this configuration for the present study, because it is best suited to understand the processes controlling metal zoning at the porphyry-epithermal transition over a lateral distance of several kilometers.

The host rock has a porosity of 0.05 and is initially saturated with pure water under a hydrostatic pressure gradient along a thermal gradient of 22.5 °C km^−1^, which is maintained by a basal heat flux of 45 mW m^−2^. During the simulation, the upper boundary represents the Earth's surface at atmospheric pressure and is permeable. Ascending fluids can discharge across the top boundary at temperatures calculated by the model. Recharging fluids enter the modeling domain as salt-free liquid water with a temperature of 10 °C. However, the fluids cannot leave the model domain via the left, right and bottom boundaries, as these are defined as no-flow boundaries. The rock properties are kept constant, including a density of 2700 kg m^−3^, a thermal conductivity of 2 W m^−1^ C^−1^, a diffusion–dispersion coefficient of 10^–12^ m^2^ s^−1^ and a heat capacity of 880 J kg^−1^ °C^−1^. The same values apply to the magma chamber, though beginning with a two-fold heat capacity that decreases gradually as the magma chamber cools considering the latent heat release during crystallization^40^.

We present results from the five most representative simulations to study the influence of three key parameters:

- Magma volume and fluid release rate: magma volume of ~ 47 km^3^ (simulation 1) or ~ 95 km^3^ (simulation 2); initial fluid concentrations of 500 ppm Cu, 330 ppm Pb, and 330 ppm Zn; without metal remobilization
- Metal contents: magma volume of ~ 47 km^3^; initial fluid concentrations of 500 ppm Cu, 33 ppm Pb, and 600 ppm Zn (simulation 3); without metal remobilization
- Remobilization: magma volume of ~ 95 km^3^ (simulation 4) or ~ 47 km^3^ (simulation 5); initial fluid concentrations of 500 ppm Cu, 330 ppm Pb, and 330 ppm Zn; with metal remobilization

## Results

### Contrasting hydrological evolutions in response to magma degassing

**Figure 2:**
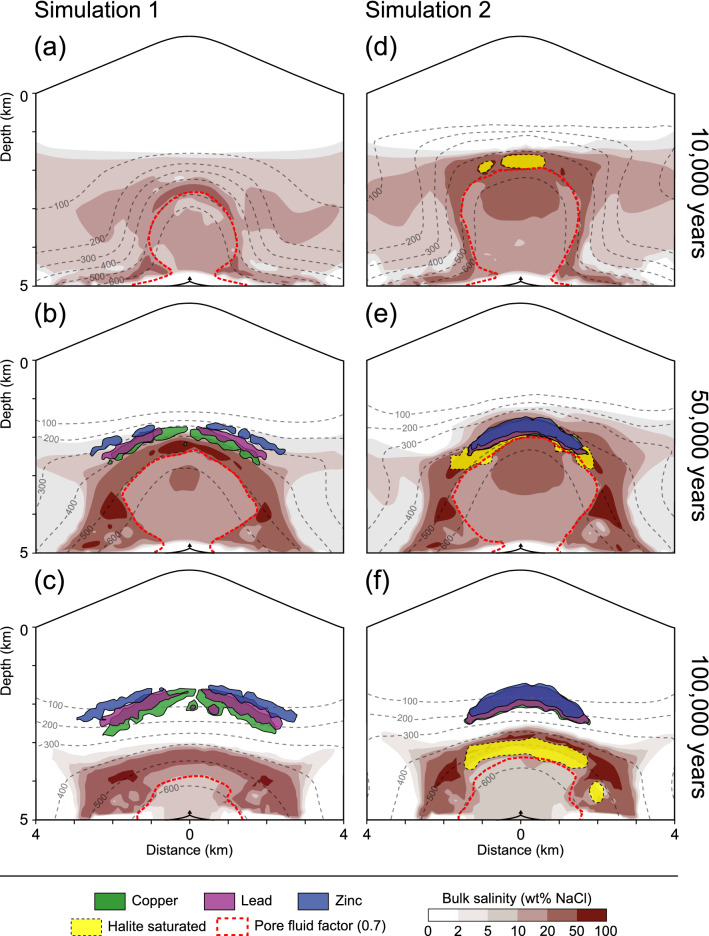
Temporal and spatial evolution of the hydrology of porphyry copper systems with a volcano topography after 10 (**a**, **d**), 50 (**b**, **e**) and 100 (**c**, **f**) kyrs of simulation time, showing modeled temperature distributions (isolines), the bulk salinity of the fluid mixture (red colors) and the region with halite-saturated fluids (yellow). Ore shells are represented by metal enrichment potentials (see text). The pore fluid factor (fluid pressure divided by lithostatic pressure) of 0.7 indicates the transition from near-hydrostatic to near-lithostatic fluid pressures. Simulation 1 used a radial factor of 0.25 and metal enrichment potentials of 250 for Cu, Zn and Pb (**a–c**). Simulation 2 used a radial factor of 0.5 and metal enrichment potentials of 500 (**d**–**f**). Arrows at a depth of 5 km refer to the fluid injection location at the cupola region of the magma reservoir.

Our simulations show that the release rate of magmatic fluids from the cupola region of a magma chamber is an important control on the outward propagation of the thermal and saline fronts as well as the location of the hydrological divide (Fig. 2). This hydrological divide (indicated by a pore fluid factor of 0.7; red dashed line) separates an inner domain dominated by ascending magmatic fluids under near-lithostatic pressures and nominally ductile rock behavior from an outer brittle domain characterized by the convection of colder fluids under near-hydrostatic pressures^23,26^. The fluid release rate is proportional to the dimensions, water content and cooling rate of the magma reservoir. In simulation 1, which assumes a reservoir volume of ~ 47 km^3^ (radial factor = 0.25), the hydrologic divide is located at a depth level of ~ 2.2 km, ~ 2.1 km and ~ 3.6 km after 10, 50 and 100 kyrs, respectively (Fig. 2a–c). In comparison, assuming a larger total reservoir volume of ~ 95 km^3^ (radial factor = 0.5) results in a hydrological divide that is located at comparatively shallower depths at ~ 1.8 km, ~ 1.9 km and ~ 3.1 km after 10, 50 and 100 kyrs, respectively (Fig. 2d–f).

In both simulations, the highest bulk salinities are found on the brittle domain side of the hydrologic divide at 10 and 50 kyrs, respectively (Fig. 2a, b, d, e). However, in simulation 1, both the fluid bulk salinity as well as the extent of the thermal and saline plume are lower compared to simulation 2 with higher magmatic fluid release (Fig. 2), reflecting contrasting phase separation behavior. During the entire degassing period of simulation 1, the primary magmatic fluid is injected as a single-phase fluid that phase-separates into a vapor and brine phase upon ascent, but no saturation with solid halite occurs. In contrast, simulation 2 rises to slightly shallower crustal levels at lower pressures where the liquid–vapor fluid mixture reaches halite saturation (yellow areas), therefore entering the three-phase (VLH) field of the H_2_O–NaCl system (Fig. 2d–f). At these conditions, the liquid phase can eventually disappear completely and the fluids enter the two-phase VH-field with a volatile, low-density vapor phase and an immobile solid halite phase, resulting in the observed high bulk salinities.

### Metal transport and precipitation

Ore precipitation in simulation 1 occurs atop the area of highest fluid bulk salinities under relatively low temperatures ranging from 400 to 200 °C (Fig. 2b, c). The ore shells are represented by a metal enrichment potential of 250 and show an outward zonation from Cu to Pb to Zn (Fig. 2b). A metal enrichment potential of 1000 corresponds to an ore grade of about 2.5 wt%, but should only be considered as a proxy as it does not include the efficiency of Cu precipitation^26^. This zonation pattern becomes more pronounced during further magma cooling (Fig. 2c). Contrastingly, ore shells in simulation 2 develop in the restricted area of halite saturation of the ore fluid, which is characterized by higher temperatures ranging from 450 to 350 °C (Fig. 2e). Ore precipitation is displayed with a metal enrichment potential of 500, taking into account that twice the amount of magmatic fluids is used in simulation 2 in comparison to simulation 1 (Fig. 2e, f). The Cu, Pb and Zn ore shells do not show a considerable zonation pattern (Fig. 2e, f). In addition to the retreat of the thermal and saline fronts caused by the cooling of the magma chamber, the area of halite saturation also recedes while the ore shells remain in place (Fig. 2c, f), showing that metal enrichment is dominated by the early phases of the hydrothermal system with the highest fluid release rates.

**Figure 3:**
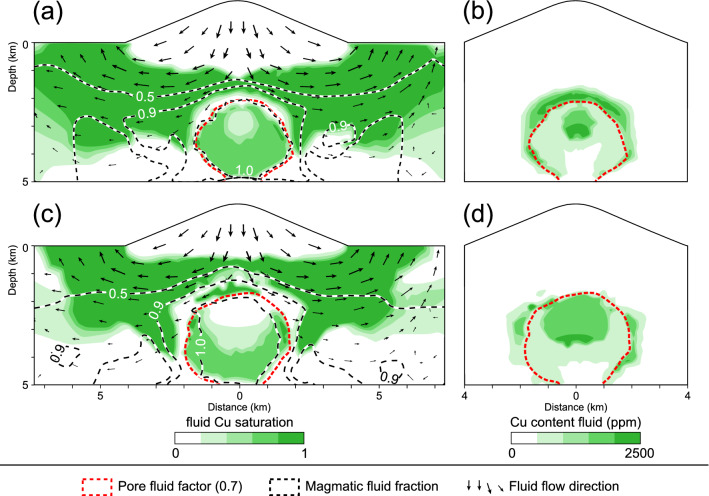
Copper saturation (**a**, **c**) and copper content (**b**, **d**) of the fluids within the hydrothermal system after 25 kyrs for the simulations 1 (**a**, **b**) and 2 (**c**, **d**). The pore fluid factor of 0.7 indicates the hydrological divide. The magmatic fluid fraction is represented by isolines of 1, 0.9 and 0.5, reflecting a magmatic fluid contribution of 100, 90 and 50% or a meteoric fluid contribution of 0, 10 and 50%, respectively. Black arrows schematically show fluid flow in the dominantly brittle domain.

The impact of varying amounts of magmatic fluids expelled from the magma chamber can further be illustrated by monitoring the degree of Cu saturation (value between 0 and 1) and the Cu content (in ppm) of the fluid (Fig. 3). The magmatic volatiles are injected as single-phase fluids with Cu contents of 500 ppm and a Cu saturation of about 0.66 at the cupola of the magma chamber at a depth of ~ 5 km (Fig. 3). With continuing ascent in the inner part (pore fluid factor > 0.7) under near-lithostatic pressures, the fluids separate into a low-salinity, low-density vapor and a high-salinity, high-density brine phase. This region is characterized by elevated bulk salinities between 30 and 50 wt% NaCl (Fig. 2b, e), low Cu saturation (Fig. 3a, c), but relatively high Cu contents (Fig. 3b, d). Even though the Cu content increases due to preferential partitioning into the brine phases, the bulk Cu saturation is reduced because metal solubilities are not linearly correlated with salinity (Fig. 1). The spatial extent of the two-phase region is smaller in simulation 1 (Fig. 3a, b) than in simulation 2 (Fig. 3b, d).

High-pressure conditions enable the further ascent of the heavy brine within the overpressured inner domain and therefore a brine-dominated zone develops atop the hydrological divide after the pressure-drop to near-hydrostatic values (Fig. 2). In simulation 2, this is the zone where halite saturation occurs, which is accompanied by a rapid increase in Cu saturation (Fig. 3c), because the saturation of the brine phase is reduced at the VLH-coexistence and can lead to the complete disappearance of the brine phase if the fluid enters the VH-field. Subsequently, Cu, Pb and Zn co-precipitate (Fig. 2e, f) and the Cu content in the ore fluid decreases (Fig. 3d). Ore precipitation occurs within magmatic fluid fractions between 0.9 and 1, reflecting a minor contribution of relatively cool, low-salinity meteoric fluids between 0 and 10% (Fig. 3c).

In the absence of halite saturation, as in simulation 1, the brine zone is enriched in Cu (Fig. 3b) and ore precipitation occurs directly atop by dilution of the metal-rich magmatic brines (Fig. 2b). This zone is characterized by the convection of significant amounts of meteoric fluids (Fig. 3a). At the time of ore precipitation, the meteoric water contribution in this simulation increases significantly to 50% in the area of the Cu and Pb shells and even to more than 50% within the Zn shell (Figs. 2b, c, 3a). Similar to simulation 2, ore precipitation in simulation 1 is accompanied by a decrease in the Cu content of the ore fluid, indicating that the majority of the metals have been deposited (Fig. 3a, b). Compared to the simulation with halite saturation (Fig. 2f), the potential ore shells have been moved to more peripheral locations and are spread out more laterally (Fig. 2c), which is less similar to the characteristic ore shell geometry of porphyry Cu deposits but approaches the maximum extent of base metal zoning observed in some porphyry-epithermal systems.

### The role of initial metal contents

**Figure 4:**
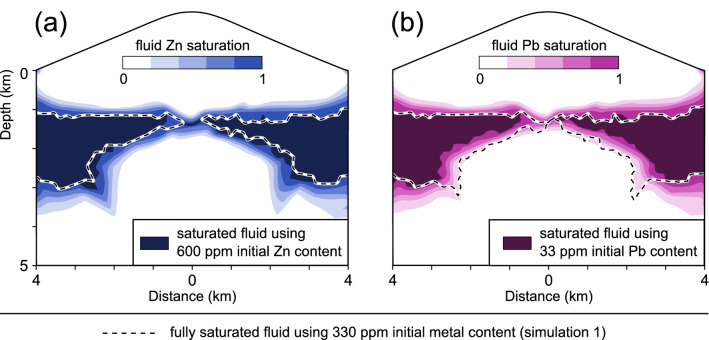
Variations in Zn (**a**) and Pb (**b**) saturations due to different initial metal concentrations in simulations 1 (dashed lines) and 3 (contours) after 25 kyrs of simulation time.

Simulation 1 produces a pattern of metal zoning which directly reflects the general trend of metal solubilities during cooling and dilution (Fig. 1), with Cu precipitating first, followed by Pb and Zn precipitating last. However, some deposits show a different zoning sequence, such as the polymetallic Morococha deposit in Peru with an outward directed Cu–Zn-Pb zonation^2^. Ore precipitation is related to the timing of saturation in the respective metals, which depends on the metal contents in the input fluid. In simulation 3, we reduce the initial Pb content to 33 ppm and increase the initial Zn content to 600 ppm. Regarding the isoline of maximum Zn saturation from simulation 1, higher Zn contents only cause minor deviations towards greater depths (Fig. 4a). In contrast, the reduced Pb contents shift precipitation towards markedly shallower depths (Fig. 4b).

**Figure 5:**
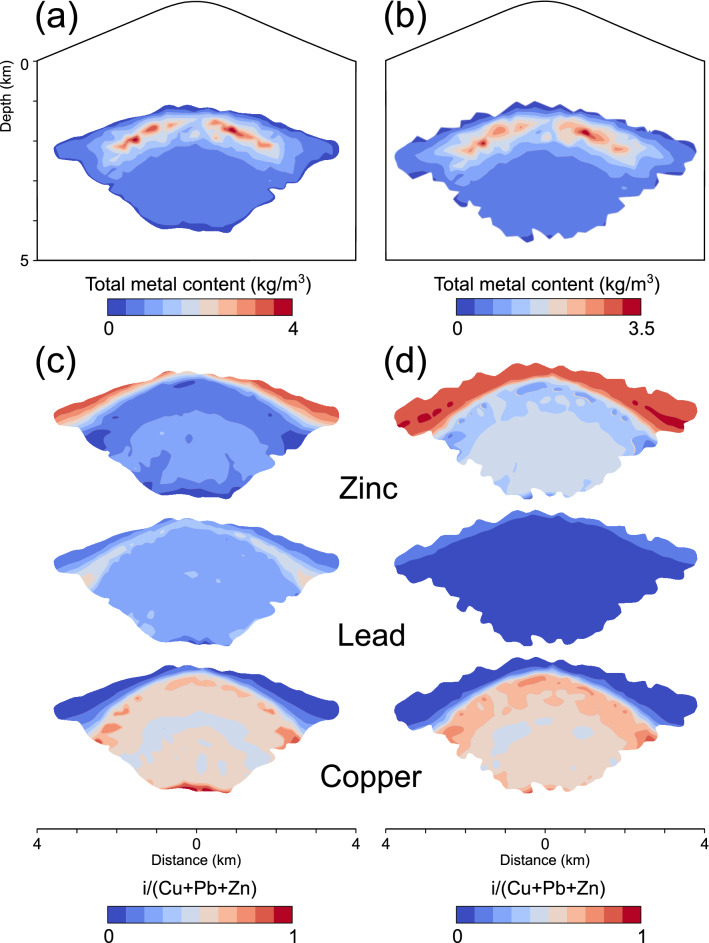
Total modeled metal content of the rock after 100 kyrs of simulation time above a threshold of 0.5 kg/m^3^ (sum of the copper, lead and zinc contents) for the simulations 1 (**a**) and 3 (**b**). Individual metal fractions within these areas of enrichment illustrate the modeled base metal zonation (**c**, **d**).

In Fig. 5a, comparison of the simulations 1 and 3 shows the effect of varying initial fluid metal contents on the final metal enrichment (after 100 kyrs of simulation time). We use an arbitrary threshold of 0.5 kg/m^3^ of the total metal contents in the rock (sum of the Cu, Pb and Zn contents) as a proxy for a potential polymetallic ore shell.

Considering initial fluid metal contents of 500 ppm Cu, as well as 330 ppm Pb and Zn (simulation 1), the highest total rock metal contents are found at the intersection of the Cu and Pb shells and thus indicate a dominance of Cu and Pb in the overall metal content of the rock (Fig. 5a). The area of the total metal contents, which is overlain by the corresponding Zn shell, however, yields rather intermediate values (Fig. 5a). In comparison, Fig. 5b presents the highest total metal contents directly on and beneath the intersection of the Cu and Zn shells and thus indicates the dominance of Cu and Zn in the overall rock metal content. The area of the total rock metal contents, which is overlain by the corresponding Pb shell, however, yields rather low values (Fig. 5b).

Figures 5c and d display the ratios of the individual metals in relation to the total metal content. Cu is found most proximal to the fluid source. Cu ratios are similar for both simulations due to the same initial Cu contents in the ore fluid and a similar overall initial metal content in the ore fluid (compare 1160 ppm for simulation 1 and 1130 ppm for simulation 3). Zinc yields relatively high ratios beyond the Cu-enriched zone (Fig. 5c, d), however, increasing initial fluid zinc contents to 600 ppm caused zinc enrichment to start at greater depths and to reach higher ratios of up to > 0.9 (dark red; Fig. 5d). In contrast, at initial fluid contents of 330 ppm, Pb has a rather moderate ratio because of the partial overlap with the Cu and Zn shells and is located proximal to the Cu zone (Fig. 5c). Initial Pb contents of 33 ppm result in low ratios located more distal to the Cu zone (Fig. 5d).

### The role of remobilization

**Figure 6:**
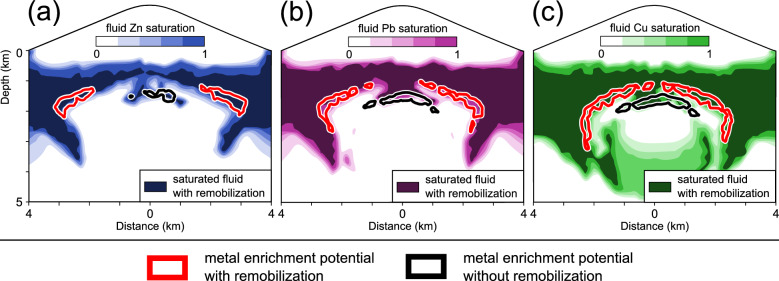
Location of Cu (**a**), Zn (**b**) and Pb (**c**) enrichment potentials of 500 after 25 kyrs of simulation time with (red isoline; simulation 4) and without (black isoline; simulation 2) metal remobilization, as well as zinc (**a**), lead (**b**) and copper (**c**) saturations of simulation 4 (color contours).

Remobilization of metals shifts the position of the ore shells beyond the brine zone to more peripheral areas (Fig. 6). Due to the downward flow of cooling ambient fluids from the stratovolcano to the flanks (see Fig. 3c), metal saturation is also moved to greater depths with remobilization (Fig. 6). The sequence of the metal zonation is similar to previous simulations, hence the Zn appearance forming the broadest shell, Pb the intermediate and the Cu shell the narrowest one (Fig. 6a–c, respectively). However, Zn and Pb develop ore shells that exclusively occupy the flanks of the upper boundary of the brine zone whereas Cu forms nearly a continuous ore shell after 25 kyrs of simulation time (Fig. 6a–c, respectively).

**Figure 7:**
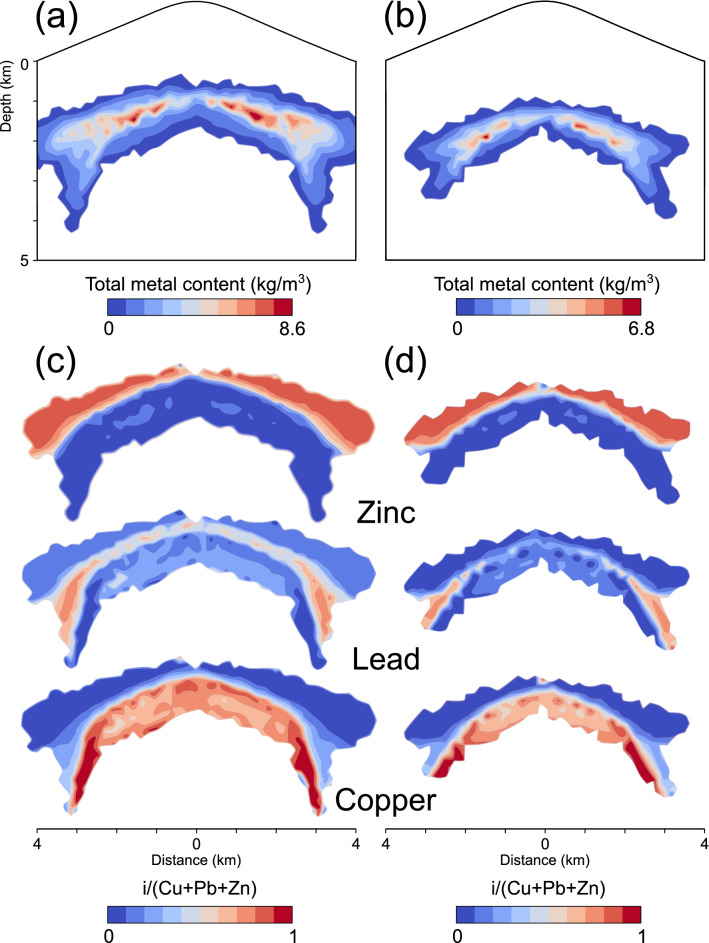
Total modeled metal content after 100 kyrs of simulation time above a threshold of 0.5 kg/m^3^ (sum of the copper, lead and zinc contents) for the simulations 4 (**a**) and 5 (**b**). Individual metal fractions within these areas of enrichment illustrate the modeled base metal zonation (**c**, **d**).

Figure 7 shows a comparison of the final shapes and extensions of the ore shells caused by remobilization for simulations with (simulation 4 with a larger magma reservoir) and without halite saturation (simulation 5 with a smaller magma reservoir) after 100 kyrs of simulation time. A threshold of > 0.5 kg/m^3^ of the total rock metal contents is displayed for simulation 4 (Fig. 7a) and 5 (Fig. 7b). Both simulations exhibit a zonation pattern from Cu to Pb to Zn. The highest total metal contents are found on and beneath the intersection of the Cu and Pb shells (Fig. 7a, b), while areas overlain by the related Zn shells yield lower values (Fig. 7a, b). The Pb and Zn ore shells are discontinuous and thickened at their outermost parts, whereas the corresponding Cu shell is continuous and rather stretched down to greater depths along the flanks (Fig. 7a). In contrast, the Zn ore shell of simulation 5 is discontinuous and peripheral precipitates are rather oriented to shallower depths, whereas the Cu and Pb shells are continuous and regularly formed (Fig. 7b).

## Discussion

The modeling results show that the release rate of the fluids originating from the magma chamber affects the location of the thermal and saline fronts (Fig. 2) and thereby fluid phase relations and mechanisms for metal precipitation. Higher fluid release rates from a larger magma reservoir move the hydrological divide to shallower depth levels and thus lower pressures where the two-phase fluid saturates in solid halite (Fig. 2d, e). In contrast, at lower fluid release rates from a smaller reservoir, meteoric fluids can cool and dilute the magmatic fluids more effectively at greater depths and higher pressures which prevents halite saturation (Fig. 2a, b).

The simulations with lower fluid release rates suggest that metals are precipitated from a two-phase vapor-brine fluid and develop zoned ore shell patterns similar to typical observations in porphyry Cu systems^1,2^ (Figs. 2b, c, 5, 7). Metal precipitation occurs directly atop the zone of highest fluid Cu contents (Fig. 3b) by admixing of relatively cool and less saline circulating meteoric water, which contributes > 10% to the overall fluid budget (Fig. 3a). Our simulations indicate that meteoric water incursion is generally necessary as a cooling and diluting fluid component for the hydrothermal systems. Progressive fluid mixing along the porphyry-epithermal transition is in line with interpretations of modern stable isotope studies, but the modelled amount of meteoric water incursion required for base metal precipitation generally exceeds their estimates^3^. The modeled sequence of base metal precipitations (Cu–Pb–Zn) follows the parameterization of metal solubilities used for this study and is also detectable in natural ore bodies^41^, especially when considering the strong overlap of the Pb–Zn mineralized zones^2,42,43^. Other porphyry-related base metal deposits show a zonation from Cu to Zn to Pb, which is more common^1,6,44^ and could be reproduced if the initial fluid is Pb-poor.

The simulations show that the metal content and solubility of the single-phase magmatic input fluid is a limiting factor controlling ore precipitation, in particular for Cu. During ascent and phase separation of the nearly Cu-saturated primary magmatic fluid, Cu contents in the bulk two-phase fluid will be considerably increased due to preferential partitioning into the brine phase and faster ascent of the vapor phase. Our modeled concentrations are comparable to values measured in brine inclusions^45^. However, this two-phase fluid is undersaturated in Cu, because solubility increases non-linearly with salinity according to data provided by Kouzmanov and Pokrovski^12^. Depressurization and cooling at the hydrological front can first increase the amount of vapor within the two-phase zone, leading to near-saturation Cu contents. However, once fluid pressures reach near-hydrostatic values, the high-density brine phase dominates, leading to Cu contents well above the ones of the input fluid, but again very low Cu saturations. Cu precipitation can thus only be reached by further cooling and/or brine dilution.

The simulations further illustrate that phase separation is a temporally and spatially continuous process, which makes the debate about whether the vapor or the brine phase transport and precipitate the bulk of the Cu ore difficult. Vapor and brine ascend together, albeit at different velocities, and are at all times fully miscible according to given TPX-dependent phase relations. Hence, even if Cu preferentially partitions into the brine phase and S into the vapor phase, both elements are likely to still be available in the two-phase mixture to form Cu sulfide minerals in case of oversaturation^18–20^.

Halite saturation can cause co-precipitation of Cu, Pb and Zn (Fig. 2e, f). Given the assumption in our model setup that halite cannot incorporate any metals, metal saturation of the residual brine subsequently increases rapidly and causes the metals to precipitate. However, the lack of significant metal zoning stands in contrast with the typical distinction between Cu-bearing ore stage veins and Pb–Zn-veins in porphyry systems. This possibly limits the potential of halite saturation of a pure magmatic fluid during ore formation. The modelled halite saturation is a transient feature during the evolution of the hydrothermal system, because halite gets redissolved by later fluid incursion. Similarly, metals temporarily stored in solid sulfide or chloride phases can develop peripheral zoning patterns during later remobilization. Such metal-rich brines could still be halite-bearing during dilution and remobilization during meteoric water incursion, which would again be permissive with an interpretation of halite-saturated ore fluids. However, whether fluid inclusions homogenizing by halite disappearance are indirect evidence for halite saturation^24^ or result from post-entrapment modifications^25^ remains debated, and our new modelling results can only confirm that it is a possible but not an inevitable ephemeral feature.

Enabling redissolution of already precipitated metals in dependence on their calculated solubility and abundance decouples halite saturation from ore precipitation and also results in the formation of a zoned ore shell pattern (simulation 4, Figs. 6, 7). Metal reprecipitation occurs beyond the brine area by further mixing with circulating meteoric water, albeit at more distal locations and at lower temperatures compared to simulations without remobilization. With the chosen simulation set-up with a stratovolcano at the surface, fluid flow in the brittle domain is directed downward and outward, which can form discontinuous ore shells (Figs. 6, 7a) with metal enrichment in peripheral areas.

**Figure 8:**
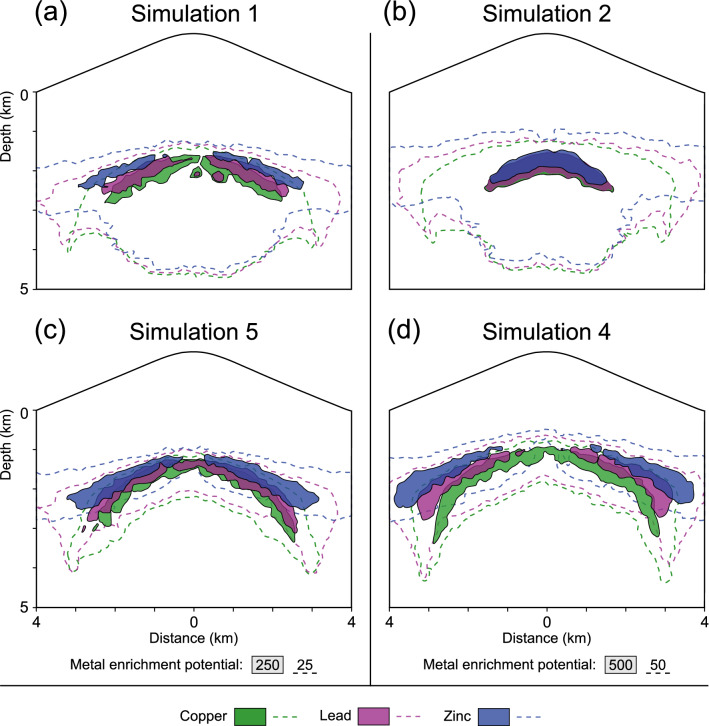
Modeled ore shells after 100 kyrs for simulations without (**a**, **b**) and with (**c**, **d**) remobilization. The different thresholds for metal enrichment potentials (25 vs. 50; 250 vs. 500) reflect the different fluid release rates controlled by the different reservoir volumes used in the respective simulations.

The simulations presented in this study produced wider and more horizontally stretched Cu ore bodies than typically observed in high-grade porphyry Cu deposits. However, previous studies have shown that limiting the extent of more vertically extended ore shells to 1–3 km can be achieved by increasing the host rock permeability in the model parametrization^23^. The dimension of the peripheral zoning with lateral extents of up to 8 km generally fits with observations of some porphyry-epithermal systems ^1,46^, in particular when also considering low-grade metal contents (Fig. 8). However, the Pb and Zn precipitation fronts do not migrate into near-surface epithermal areas even with the consideration of remobilization (Fig. 8a, c, d), therefore displaying typical sub-epithermal veins^1^. Distinct epithermal mineralization events may thus require a distinct fluid event or pulse that could lead to higher fluid temperatures in the epithermal regime.

Our simulations share many similarities with the simulations of Blundy, et al.^22^, but diverge in the residence times of metal-enriched magmatic brines formed by phase separation. Whereas Blundy, et al.^22^ simulate long-lived brine lenses and propose an economic potential for metal extraction, our simulations suggest a rather transient occurrence of hypersaline brine layers that become diluted by meteoric water incursion and transported out of the system. Fluid mixing is enhanced in our model, because it includes a magma reservoir at 5 km depth as a driver for external convection, whereas Blundy et al.^22^ single out the effect of magmatic volatile injection. The two models also use different parameterizations of the host rock permeability. Hence, the residence times of magmatic brines may depend on permeability evolution and the size and depth of the underlying magma reservoir—parameters that so far are typically not well constrained in both fossil and active systems.

The modeled physical hydrology and its geochemical implications rely on a number of simplifications, with some being intentional to reduce the complexity of the system while others are due to a lack of adequate data or numerical tools. Our modeling efforts are still restricted by limitations in the formulation of physical and chemical processes, e.g. the dynamic permeability evolution at brittle and ductile conditions, fluid-rock reactions influencing variations in pH, redox conditions, sulfide availability, mineral chemistry and species of aqueous fluids which can have a significant effect on metal solubilities^12^. Lowering the pH value from 5 to 4, for example, may increase the metal contents and solubilities of the single-phase fluid by one order of magnitude for Cu and two orders of magnitudes for both Pb and Zn^12^. Such complexities will only be resolvable once full reactive-transport modeling for magmatic-hydrothermal systems should become possible. However, our simulations can already be used to gain new insights into the feasibility of ore formation by cooling and dilution of magmatic fluids.

## Conclusions

We investigated the role of phase separation, brine formation and remobilization on base metal precipitation in porphyry Cu systems using an advanced numerical model that incorporates published temperature- and salinity-dependent metal solubilities. Based on these simplified parameterizations, the simulations suggest some first-order feedbacks between physical hydrology and geochemistry:

1. Our simulations can reproduce the general pattern of metal zoning with lateral extents of up to several kilometers and Pb–Zn mineralization being peripheral to Cu-mineralization for scenarios that avoid halite saturation by cooling and dilution of magmatic brines due to significant incursion of meteoric water.
2. The vapor and brine phases of the two-phase ore-stage fluid ascend together with different residence times, but still in contact with each other at all times. The brine phase becomes enriched in base metals due to preferential partitioning, but remains metal-undersaturated even for saturated primary single-phase input fluids if solubilities increase non-linearly with fluid salinities.
3. Our simulations were able to produce distinct ore shells, especially when the model setups accounted for potential remobilization of metals followed by reprecipitation. Saturation of solid halite can lead to high-grade deposits with simultaneous precipitation of ore metals, which is in contrast with characteristic vein types at the deposits, but therefore emphasizes the importance of remobilization for future modeling approaches.
4. Brine lenses underneath stratovolcanoes are transient features, leading to direct ore formation rather than longer-term storage of a metal-rich brine phase.
5. Future simulations should include improved thermodynamic models, comprising pH, redox, chloride and bisulfide complexing to provide more detailed insight into the relevant geochemical processes including the role of fluid-rock reactions.

## Data Availability

The study uses the software CSMP +  + and the algebraic multi-grid solver SAMG, which are subject to licensing via https://mineralsystems.ethz.ch/software/csmp.htmland https://www.scai.fraunhofer.de/de/geschaeftsfelder/schnelle-loeser/produkte/samg.html. The necessary information on the numerical method and the simulation setups is provided in the methods section and the references therein (Weis, 2015; Weis et al., 2012, 2014).
