# Author Correction: Hydrological controls on base metal precipitation and zoning at the porphyry-epithermal transition constrained by numerical modeling

**DOI:** 10.1038/s41598-023-34496-y

**Published:** 2023-05-08

**Authors:** Malte Stoltnow, Philipp Weis, Maximilian Korges

**Affiliations:** 1 Institute of Earth and Environmental Science, University of Potsdam, Karl-Liebknecht-Straße 24/25, 14476 Potsdam, Germany; 2 GFZ German Research Centre for Geosciences, Telegrafenberg, 14473 Potsdam, Germany

Correction to: *Scientific Reports* https://doi.org/10.1038/s41598-023-30572-5, published online 07 March 2023

The original version of this Article contained an error in the Methods, under the subheading ‘Governing equations’, where the notation of Equation 6 was incorrect.

$$\mathrm{log}\,({C}_{i})={a}_{00,i}+{a}_{10,i}T+{a}_{01,i}\,\mathrm{ln}\,(x)+{a}_{20,i}{T}^{2}+{a}_{11,i}\,\mathrm{ln}\,(x)T$$

now reads:

$$log({C}_{i})={a}_{00,i}+{a}_{10,i}T+{a}_{01,i}x+{a}_{20,i}{T}^{2}+{a}_{11,i}xT$$

The original Article has been corrected.

